# Supplementary material for: A phosphoramidate modification of FUDR, NUC-3373, causes DNA damage and DAMPs release from colorectal cancer cells, potentiating lymphocyte-induced cell death
Source: PLoS One. 2025 Sep 16;20(9):e0331567. doi: 10.1371/journal.pone.0331567 (PMC12440158; doi:10.1371/journal.pone.0331567)
Supplement: S1 Table — Abbreviations: UC – unconjugated; WB – western blot; IHC – immunohistochemistry; IF – immunofluorescence; FC – flow cytometry; AB – antigen blocking. (DOCX) [file pone.0331567.s001.docx]

| **Antibody/Target** | **Conjugate** | **Application** | **Supplier** | **Catalogue no.** |
| --- | --- | --- | --- | --- |
| β-Actin | UC | WB (1:2,000) | Cell Signaling Technology | 3700s |
| Donkey anti-rabbit | IRDye 800RD | WB (1:10,000) | Licor | 926-32213 |
| Donkey anti-mouse | IRDye 680RD | WB (1:10,000) | Licor | 926-68072 |
| HMGB1 | UC | IF | Abcam | 18256 |
| γ-H2AX | UC | IF | Cell Signalling Technology | 9718 |
| Hsp-70 | UC | FC (2.5 μg/ml) | Biolegend | 648002 |
| Goat anti-mouse | FITC | FC (1:10,000) | Abcam | 6785 |
| CRT | UC | FC (1:800) | Cell Signaling Technology | 12238 |
| Goat anti-rabbit | AlexaFluor 488 | FC (1:10,000) | Cell Signaling Technology | 4412 |
| CD17a (LAMP1) | AlexaFluor 488 | FC (5 μl/1 x 10e6 cells) | Biolegend | 328610 |
| CD56 | AlexaFluor 647 | FC (5 μl/1 x 10e6 cells) | Biolegend | 318314 |
| TIGIT | PE | FC (5 μl/1 x 10e6 cells) | Biolegend | 373703 |
| IFN-γ | AlexaFluor 488 | FC (5 μl/1 x 10e6 cells) | Biolegend | 505813 |
| CD274 (PD-L1) | FITC | FC (5 μl/1 x 10e6 cells) | Biolegend | 374510 |
| CD3 | UC | Stim (1 μg/ml) | Biolegend | 300302 |
| CD28 | UC | Stim (1 μg/ml) | Biolegend | 302902 |
| Cytokeratin 7/8 | AlexaFluor 647 | FC (5 μl/1 x 10e6 cells) | BD Biosciences | 563614 |
| HLA-DR | Pacific Blue | FC (5 μl/1 x 10e6 cells) | Biolegend | 307633 |
| Nivolumab (anti-PD-1) | UC | AB (10 μg/ml) | Selleckchem | A2002 |

**Supplementary Table 1: Antibodies used**

Abbreviations: **UC** – unconjugated; **WB** - western blot; **IHC** – immunohistochemistry; **IF** – immunofluorescence; **FC** – flow cytometry; **AB** – antigen blocking.
