# Supplementary material for: A phosphoramidate modification of FUDR, NUC-3373, causes DNA damage and DAMPs release from colorectal cancer cells, potentiating lymphocyte-induced cell death
Source: PLoS One. 2025 Sep 16;20(9):e0331567. doi: 10.1371/journal.pone.0331567 (PMC12440158; doi:10.1371/journal.pone.0331567)
Supplement: S1 Methods — HCT116/PBMC cocultures were set up in 6-well plates as previously described with the amount of PBMCs added after drug incubation being 1 x 10^6^ cells. RNA lysates were prepared at 24 and 48 hours coculture time points using a RNeasy Kit (Qiagen) as per manufacturer’s instructions. Extracted RNA was then converted to cDNA using a QuantiTect Reverse Transcription Kit (Qiagen). Primers for qPCR included TNF-α: fwd- GCTGCACTTTGGAGTGATCG, rev- GCTGAGGGTTTGCTACAA; IFN-γ: fwd- CGTTTTGGGTTCTCTTGGCT, rev- TTTCTGTCACTCTCCTCTTTCC; IL-2: fwd- TTACATGCCCAACAAGGCCA, rev- TGGTTGCTGTCTCATCAGCAT; and PD-L1: fwd- AGGCCGAAGTCATCTGGACAAG, rev- TCCTCTCTCTTGGAATTGGTG (ThermoFisher) and QuantiTect Primer Assays for GAPDH (Qiagen) and β-Actin (Qiagen). Samples for qPCR were prepared using QuantiNova SYBR green PCR Kit (Qiagen) and run on a Rotor-Gene Q (Qiagen) and associated software. Ct data was exported to and analysed in Excel using the ∆∆Ct method. (PDF) [file pone.0331567.s002.pdf]

Supplementary Methods:

Gene expression of cytokines and PD-L1

HCT116/PBMC cocultures were set up in 6-well plates as previously described with the amount of PBMCs added after drug incubation being 1 x 10^6 cells. RNA lysates were prepared at 24 and 48 hours coculture time points using a RNeasy Kit (Qiagen) as per manufacturer's instructions. Extracted RNA was then converted to cDNA using a QuantiTect Reverse Transcription Kit (Qiagen). Primers for qPCR included TNF-α: fwd- GCTGCACTTTGGAGTGATCG, rev- GCTGAGGGTTTGCTACAA; IFN-γ: fwd- CGTTTTGGGTTCTCTTGGCT, rev- TTTCTGTCACTCTCCTCTTTCC; IL-2: fwd- TTACATGCCCAACAAGGCCA, rev- TGGTTGCTGTCTCATCAGCAT; and PD-L1: fwd- AGGCCGAAGTCATCTGGACAAG, rev- TCCTCTCTCTTGGAATTGGTG (ThermoFisher) and QuantiTect Primer Assays for GAPDH (Qiagen) and β-Actin (Qiagen). Samples for qPCR were prepared using QuantiNova SYBR green PCR Kit (Qiagen) and run on a Rotor-Gene Q (Qiagen) and associated software. Ct data was exported to and analysed in Excel using the ΔΔCt method.
