# Supplementary material for: A phosphoramidate modification of FUDR, NUC-3373, causes DNA damage and DAMPs release from colorectal cancer cells, potentiating lymphocyte-induced cell death
Source: PLoS One. 2025 Sep 16;20(9):e0331567. doi: 10.1371/journal.pone.0331567 (PMC12440158; doi:10.1371/journal.pone.0331567)
Supplement: S1 Fig — Flow cytometry data for Hsp-70 on HCT116 (top) and SW480 (bottom) cells after being exposed to vehicle control (DMSO – left) or 10 µM NUC-3373 (right) for 24 hours. Gates drawn based on unstained controls. (PDF) [file pone.0331567.s003.pdf]

Supplementary Figures:

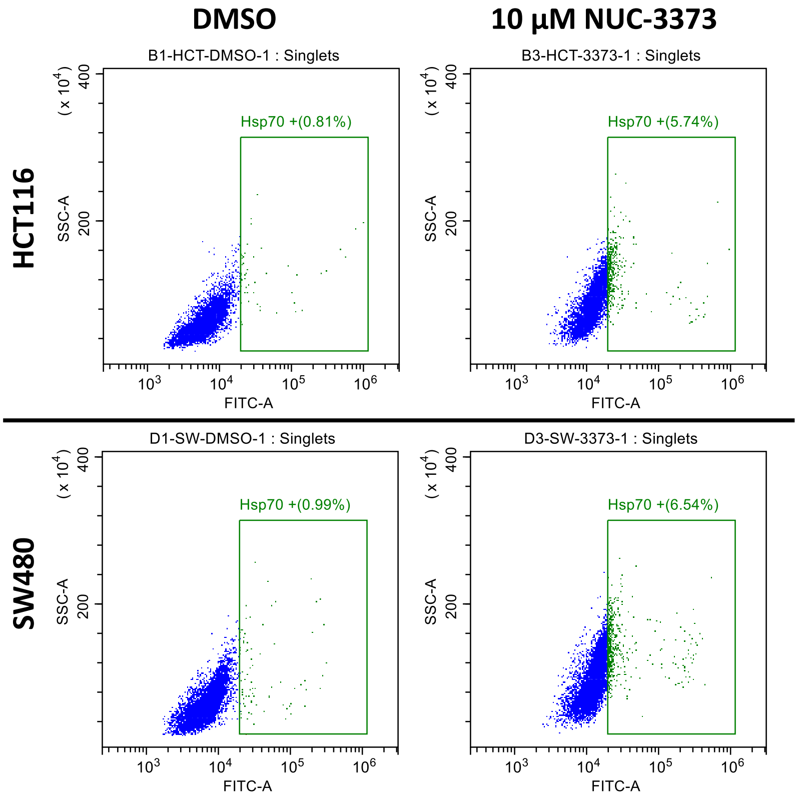

Fig S1. NUC-3373 causes increased surface expression of Hsp-70 on CRC cells: Flow cytometry data for Hsp-70 on HCT116 (top) and SW480 (bottom) cells after being exposed to vehicle control (DMSO – left) or 10 µM NUC-3373 (right) for 24 hours. Gates drawn based on unstained controls.
