# Supplementary material for: A phosphoramidate modification of FUDR, NUC-3373, causes DNA damage and DAMPs release from colorectal cancer cells, potentiating lymphocyte-induced cell death
Source: PLoS One. 2025 Sep 16;20(9):e0331567. doi: 10.1371/journal.pone.0331567 (PMC12440158; doi:10.1371/journal.pone.0331567)
Supplement: S2 Fig — Flow cytometric analysis of NK-92 MI cells cocultured with HCT116 (A. & B.) or SW480 cells (C. & D.) that had been pre-treated with either DMSO control (light grey), 10 µM oxaliplatin (yellow), or 10 µM NUC-3373 (blue). Histograms and plots display surface staining of LAMP1 (A. & C.) and intracellular staining of IFN-γ (B. & C.). Bar charts show MFI fold change normalised to that of the DMSO control. Oxaliplatin (Sigma Aldrich) was dissolved in deionised water to a stock concentration of 10 mM. (PDF) [file pone.0331567.s004.pdf]

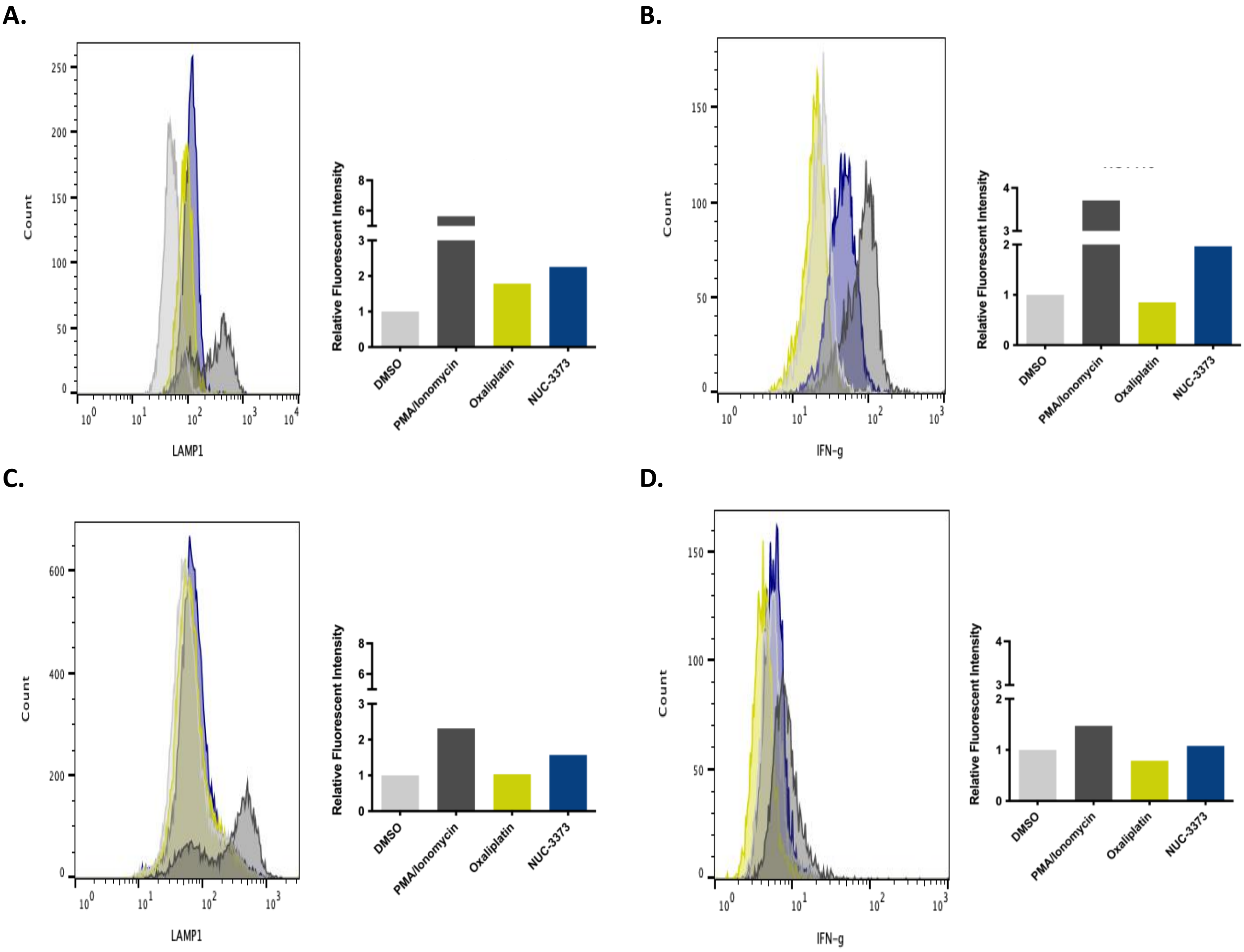

Fig S2. LAMP1 and IFN-γ induction in NK-92 MI cells after coculture with HCT116 and SW480 cells pre-treated with NUC-3373 and oxaliplatin: Flow cytometric analysis of NK-92 MI cells cocultured with HCT116 (**A. & B.**) or SW480 cells **(C. & D.)** that had been pre-treated with either DMSO control (light grey), 10 µM oxaliplatin (yellow), or 10 µM NUC-3373 (blue). Histograms and plots display surface staining of LAMP1 (A. & C.) and intracellular staining of IFN-γ (B. & C.). Bar charts show MFI fold change normalised to that of the DMSO control. Oxaliplatin (Sigma Aldrich) was dissolved in deionised water to a stock concentration of 10 mM.
