# Supplementary material for: A phosphoramidate modification of FUDR, NUC-3373, causes DNA damage and DAMPs release from colorectal cancer cells, potentiating lymphocyte-induced cell death
Source: PLoS One. 2025 Sep 16;20(9):e0331567. doi: 10.1371/journal.pone.0331567 (PMC12440158; doi:10.1371/journal.pone.0331567)
Supplement: S3 Fig — HCT116 cells were pre-treated with either 10 µM NUC-3373 or DMSO for 24 hours before coculture with patient-derived PBMCs with or without stimulation with anti CD3/28. A. Representative gene expression of IL-2, TNF-α, IFN-γ, and PD-L1 in cocultures was determined by qPCR at 24hr. Gene expression plotted as fold change relative to 24 hours DMSO control. qPCR data was analysed using the ∆∆Ct method. B. Flow cytometry data for PBMC/HCT116 cells stained for cell surface PD-L1 at 48 hours of coculture. Additional staining of intracellular pan-cytokeratin was used to differentiate HCT116 from PBMC. (PDF) [file pone.0331567.s005.pdf]

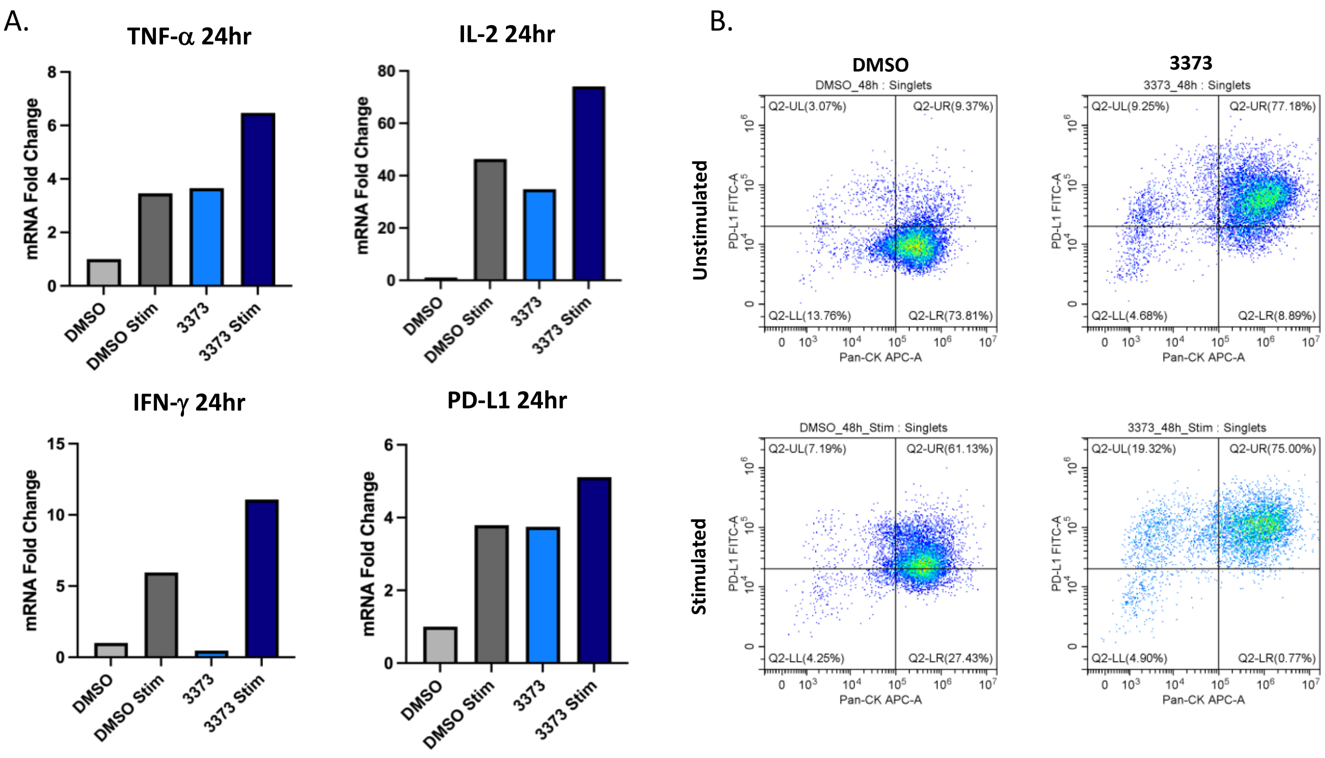

Fig S3. NUC-3373 pre-treatment increases expression of cytokines and PD-L1 in PBMC/HCT116 cocultures: HCT116 cells were pre-treated with either 10 μM NUC-3373 or DMSO for 24 hours before coculture with patient-derived PBMCs with or without stimulation with anti CD3/28. **A.** Representative gene expression of IL-2, TNF-α, IFN-γ, and PD-L1 in cocultures was determined by qPCR at 24hr (n=1). Gene expression plotted as fold change relative to 24 hours DMSO control. qPCR data was analysed using the ΔΔCt method. **B.** Flow cytometry data for PBMC/HCT116 cells stained for cell surface PD-L1 at 48 hours of coculture. Additional staining of intracellular pan-cytokeratin was used to differentiate HCT116 from PBMC (n=1).
