# Supplementary figures and images for: A phosphoramidate modification of FUDR, NUC-3373, causes DNA damage and DAMPs release from colorectal cancer cells, potentiating lymphocyte-induced cell death
Source: PLoS One. 2025 Sep 16;20(9):e0331567. doi: 10.1371/journal.pone.0331567 (PMC12440158; doi:10.1371/journal.pone.0331567)

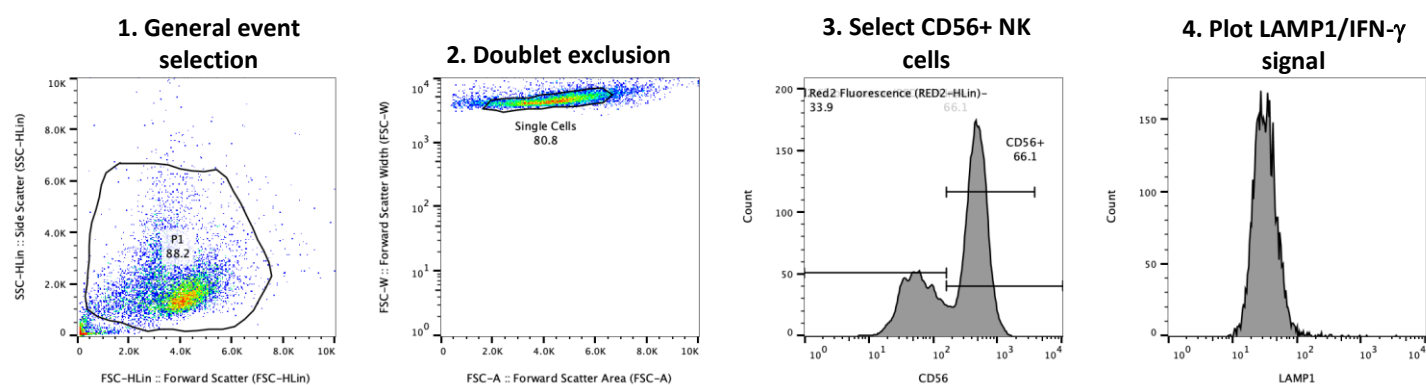

**Fig S4.** Flow cytometry gating strategy used for assessment of LAMP1 expression on CD56+ NK cells.

Supplement: S4 Fig — (PDF) [file pone.0331567.s006.pdf]
