# Supplementary material for: A phosphoramidate modification of FUDR, NUC-3373, causes DNA damage and DAMPs release from colorectal cancer cells, potentiating lymphocyte-induced cell death
Source: PLoS One. 2025 Sep 16;20(9):e0331567. doi: 10.1371/journal.pone.0331567 (PMC12440158; doi:10.1371/journal.pone.0331567)
Supplement: S7 Fig — (PDF) [file pone.0331567.s009.pdf]

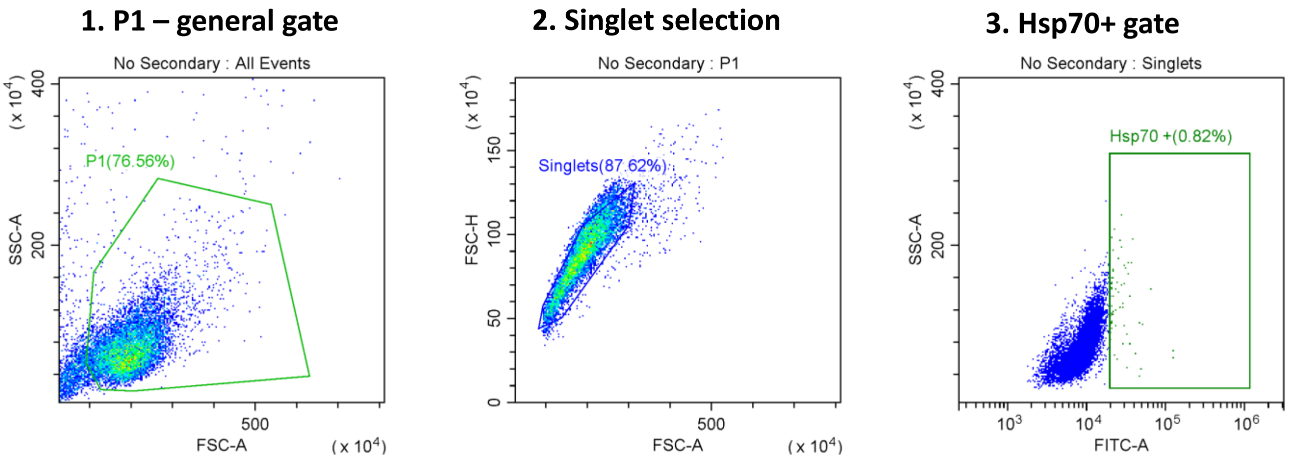

Fig S7. Flow cytometry gating strategy used for assessment of Hsp-70 surface expression on CRC cells.
